# Cerebrospinal fluid outflow along lumbar nerves and possible relevance for pain research: case report and review

**DOI:** 10.3325/cmj.2014.55.399

**Published:** 2014-08

**Authors:** Karl Bechter, Bernd Schmitz

**Affiliations:** 1 Department of Psychiatry and Psychotherapy II, Ulm University/Bezirkskrankenhaus Guenzburg, Guenzburg, Germany; 2 Department of Neuroradiology, Ulm University/Bezirkskrankenhaus Guenzburg, Guenzburg, Germany

## Abstract

CSF outflow through the cribriform plate near the olfactory nerves and the outflow along brain and spinal nerves are together known as peripheral CSF outflow pathway (PCOP). It is still not clear whether the PCOP has pathogenetic relevance. Our previous clinical observations have indicated that CSF may interact with nerves along the PCOP and in this article we present our finding of CSF outflow demonstrated by myelography in a single patient. We also discuss unexplained experimental pain pathomechanisms against the background of the PCOP hypothesis. We observed that CSF flowed along lumbar nerves in distal direction at a speed of about 10 cm per hour on its way through the tissues, mainly muscles. Total CSF outflow volume at the lumbar site was remarkable. CSF outflow at lumbar nerves was also documented by neuroradiology. It is plausible that CSF signaling serves for interaction with nerves along the PCOP, which could explain previously unknown pathomechanisms in pain generation. Experimental findings of tactile pain hypersensitivity within lumbosacral pain pathways could be explained by releasing of molecules, microparticles, or exosomes into the CSF by mast cells, which then move with CSF outflow along the PCOP and interact with nerves, initiating even retrograde synaptic stripping.

## INTRODUCTION: THE PERIPHERAL CSF OUTFLOW PATHWAYS (PCOP)

It has been traditionally accepted that cerebrospinal fluid (CSF) flows from the subarachnoid spaces (SAS) through the cribriform plate into the cervical lymphatics, and similarly, along all brain nerves and all peripheral nerves into the respective tissues. This CSF outflow can transport antigens from CSF spaces to the extracellular fluid of peripheral tissues, which are then collected into the lymphatic system (1). CSF outflow through the cribriform plate to cervical lymphatics has been shown to be important for CNS-specific immune responses (2,3), but CSF outflow along (other) brain nerves and spinal nerves has been only poorly investigated. Our previous research investigated low level neuroinflammation (LLNI) subgroups in severe psychiatric disorders (4). Affective and schizophrenia spectrum disorders may be accompanied by pain and other sensory symptoms (5), which could be explained at least in part by LLNI pathomechanisms that arise from an interaction between CSF and peripheral nerves along the PCOP. It is also suggested that PCOP-associated symptoms prevail in classic neuroinflammation and possibly even in systemic inflammation (6). CSF has been found to have an important signaling function; CNS volume transmission involves signaling within the CNS extracellular spaces with a link to CSF signaling (7).

## THE PCOP HYPOTHESIS

We performed CSF filtration as experimental therapy in cases of severe therapy resistant depression or schizophrenia, with the hypothesis that LLNI states may be responsible for therapy resistance (8). During such experimental CSF filtration procedures performed daily over five days via lumbar intrathecal catheter, we observed a surprisingly rapid improvement of even chronic pain (9-11). In such cases, the CSF analysis showed LLNI to be present in the SAS. Thus, we assumed that chronic pain resulted from an interaction between pathogenic CSF contents and the spinal cord and/or spinal nerves roots within the SAS. There however remained specific clinical details to be explained: for example, in some cases pain perception showed waxing-waning asymmetries and was accompanied by slight paresis, both symptoms being prevalent over months. These specific aspects cannot be explained by interactions of pathogenetic CSF and nervous system exclusively within the SAS, as this would likely elicit symmetric appearance of symptoms. Thus, the idea was that CSF signaling came from more distal sites, meaning PCOP (9). Based on such observations and on extensive literature, a detailed PCOP hypothesis was formulated (6), proposing a complex scenario of interactions between CSF and potentially all parts of the CNS that are in contact with the CSF. This would include the hypothetic CSF signaling on brain nerves and peripheral nerves along the PCOP through the tissues, including free nerves ends, where CSF outflow winds up. In conditions with abnormal CSF content, like in LLNI states or in classical neuroinflammation, such interactions at the PCOP sites might cause local dysfunctions and symptoms. However these symptoms might not be easily detected, as the PCOP is distributed throughout the whole body, and might be interpreted to be coming from blood, as blood is a ubiquitous signaling system. Thus, PCOP associated symptoms could be differentiated from blood-associated symptoms only by minor local typological differences. Such CSF signaling-related symptoms may be waxing-waning symptoms, slight asymmetries, or different distribution of symptoms in larger body parts related to changes of posture with presumed associated CSF outflow variations (6).

## PCOP HYPOTHESIS AS A POSSIBLE EXPLANATION FOR WIDESPREAD TACTILE PAIN HYPERSENSITIVITY

Levy et al (12) investigated widespread tactile pain hypersensitivity from activated trigemino-cervical and lumbosacral pain pathways induced by mast cell degranulation, but it remained unsolved what mechanism mediated hind paw hypersensitivity; for example with the available hypothesis it was difficult to explain the details of the time course of the changes. We would like to contribute to these observations by proposing a following scenario involving the PCOP.

1. In general, CSF signaling can be mediated by solutes, especially proteins, cells, exosomes, microvesicles, or microparticles containing powerful signaling molecules (7,13).

2. CSF flows from the SAS along the PCOP between the epineurium and perineurium, making its way down (or distally from the SAS) the nerves all along its course touching also the neuronal ganglia. It eventually reaches the peripheral tissues at the area where nerves end and CSF outflow winds up to join the extracellular tissue fluid.

3. Any pathogenic CSF contents might potentially interact with nerves at all PCOP sites where such interaction is possible, causing pathogenetic consequences at different sites from the SAS to the nerve ends, and even to the neighboring tissues near nerve ends.

4. Pathogenic CSF contents may for example be released to the SAS from mast cells within the meninges near the SAS. Molecules and particles released by mast cell degranulation could then reach through CSF outflow all PCOP sites and elicit local effects:

a) At peripheral neuronal ganglia the pathogenic CSF contents may easily elicit retrograde synaptic stripping at the spinal cord level and thus contribute to pain hypersensitivity generation (personal communication with Georg Kreutzberg) (14, see references 29,30 in 14).

b) In the experiments by Levy et al (12), the mast cell degranulation took place in many tissues and at many sites including the meninges (dura), but was especially strong near free nerve ends. Molecules released into both, the SAS or near free nerves ends, could act together. Also locally released signaling molecules (from mast cells or others) might gain new effector qualities at local sites when they interact with CSF, or CSF signaling could influence locally residing cells, which could then become activated to enhance other pathogenic downstream mechanisms.

c) Unfortunately the CSF outflow patterns and local volumes at various parts of the PCOP appear to be rather speculative. CSF outflow may depend on the posture (6) and experiments with absorption of radioactive tracers showed that it also depended on motor activity, when it was nearly doubled (15). These authors interpreted the disappearance of tracer from the SAS as absorption, but they could not differentiate absorption from outflow, because of the technical approach they used. Edsbagge et al (15) instead clearly demonstrated CSF outflow (see for comparison the evident considerable CSF outflow volume along lumbar nerves during routine myelography in our case).

d) CSF outflow and signaling by solutes along the PCOP may be accompanied by trafficking (and signaling) of CSF cells along the PCOP. CSF cell trafficking along the PCOP was proven at the cribriform plate, after which it reached the cervical lymphatics (2,16,17). It is plausible that CSF cell trafficking may similarly take place in all other parts of the PCOP, that is, brain nerves and spinal nerves, but there is still a lack of evidence to confirm this. We have recently demonstrated in a single case that leukemia cells followed the PCOP from the subarachnoid spaces along lumbar nerves found most distally in subcutaneous tissues (18). Thus, CSF cells in general may have the potential of trafficking along the PCOP and possibly contributing to local pathogenic or protective mechanisms at the PCOP.

## PHYSIOLOGICAL CSF OUTFLOW AT LUMBAR NERVES IN HUMANS DEMONSTRATED BY NEURORADIOLOGICAL APPROACH

**Figure 1:**
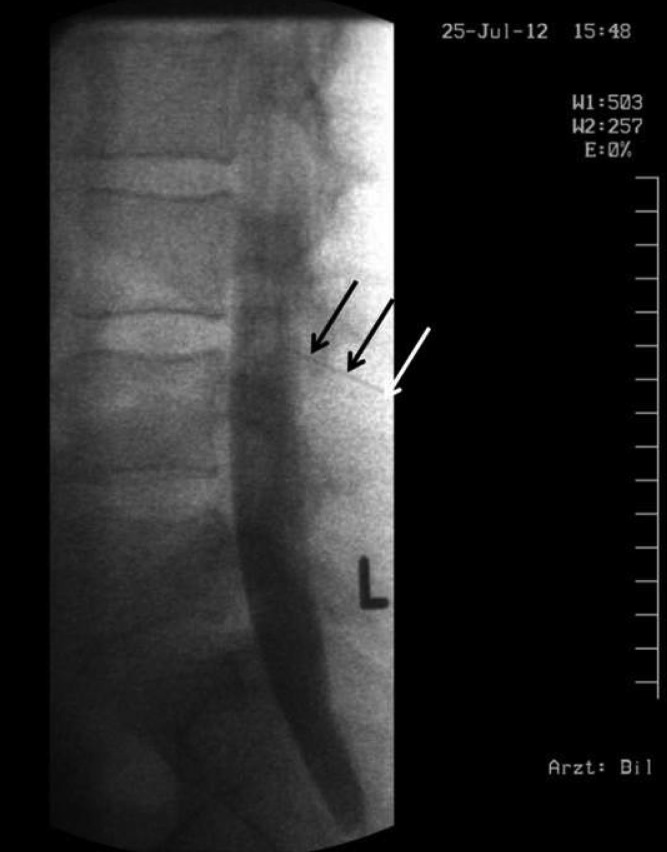
Lateral view of the spine with the needle inserted into the lumbar subarachnoid spaces. The water soluble contrast agent (15 mL Solutrast 250M) was just injected and distributed immediately throughout the lumbar subarachnoid space (in black). Time 15:48.

**Figure 2:**
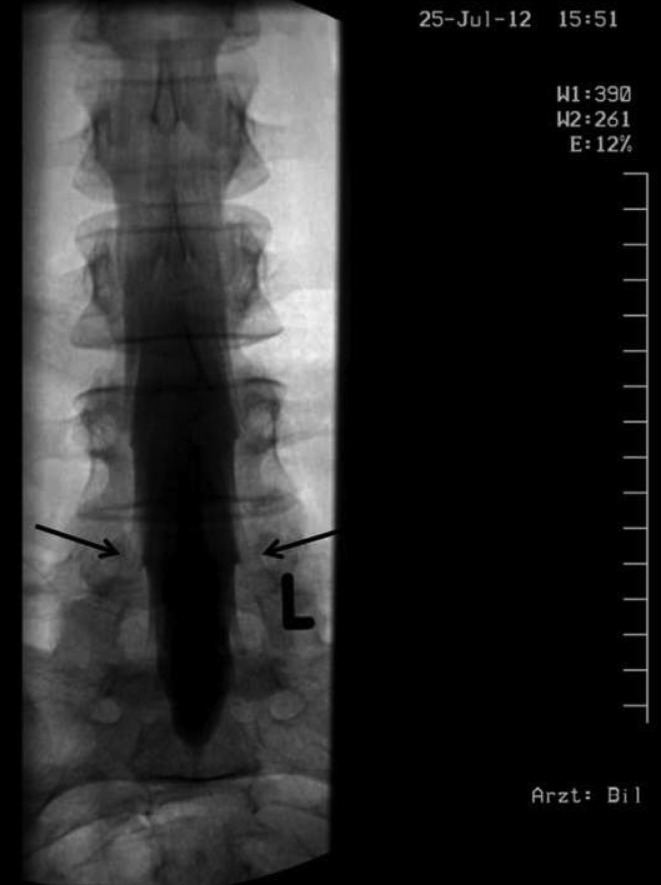
Posterior-anterior view of the spine (L = left) filling the lumbar subarachnoid space (black). The contrast agent has filled both sides (arrows) and the nerve root sleeves, which demonstrate part of the subarachnoid spaces. Time 15:51.

**Figure 3:**
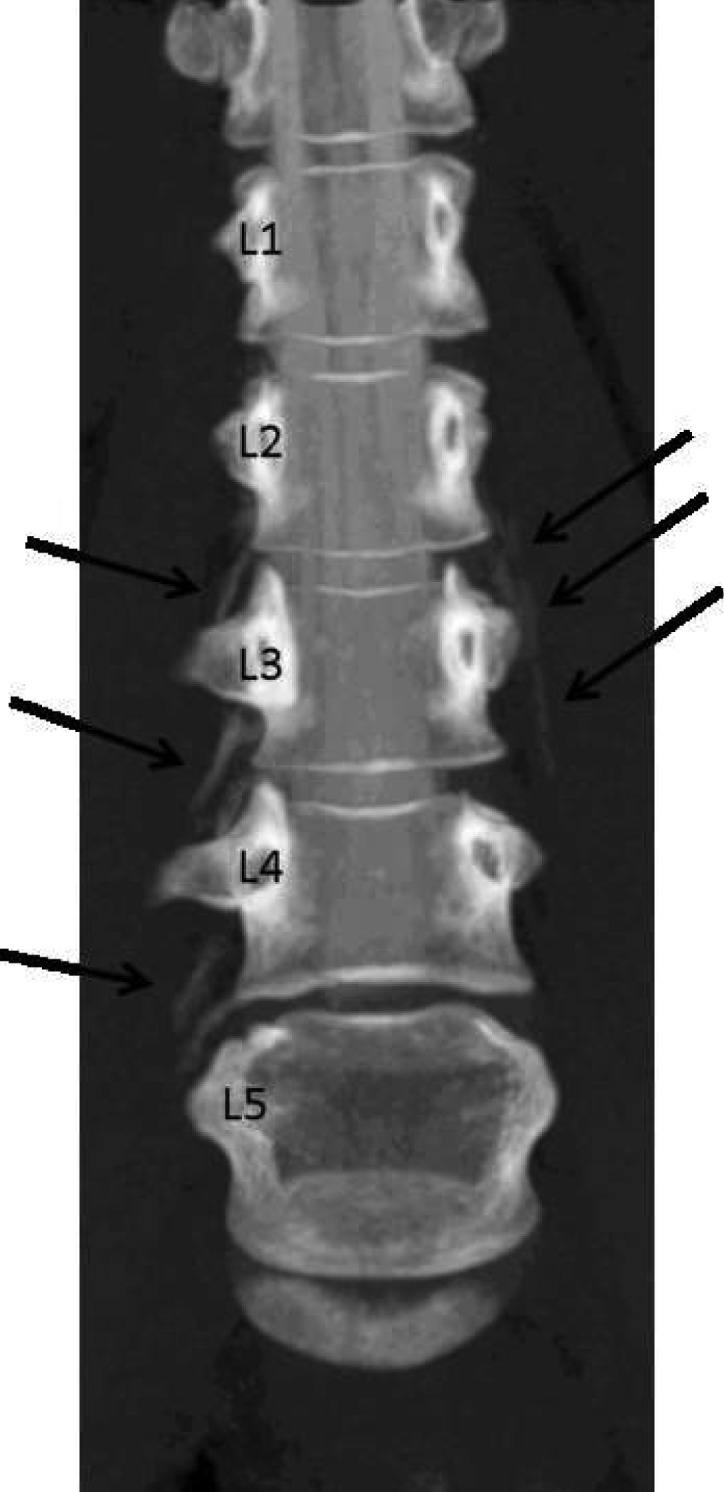
After injection of the contrast agent, CT scan was performed in prone position on a Philips Brilliance 40 CT-Scanner (Hamburg, Germany) in spiral mode, collimation 40 × 0.625 mm, 120 kV, 110 mAs. Multiplanar reconstructions were calculated. Time 16:13. Maximum intensity projection in anterior-posterior view showing the lumbar spine (lumbar vertebral bodies 1-5, see numbers). The contrast agent is now visible on both sides outside the subarachnoid space distributing parallel to the spinal nerves.

**Figure 4:**
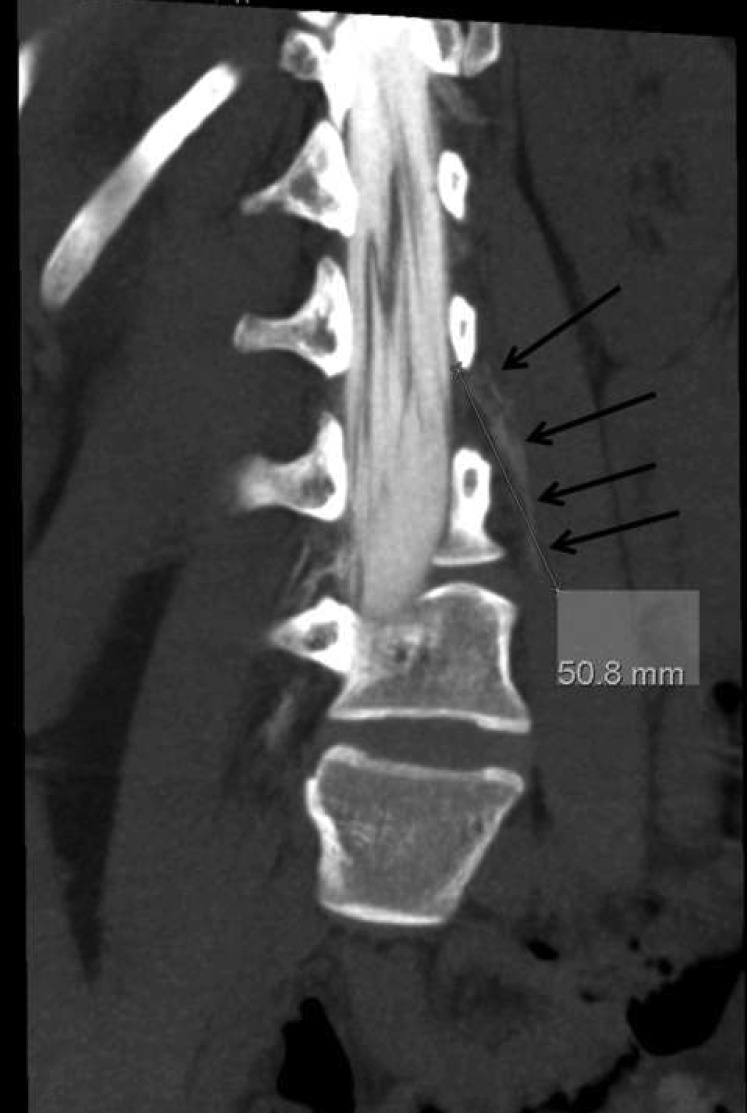
Oblique view of the spinal nerve parallel to the natural course of the nerve through the tissue. The contrast agent made a distance of 50.8 mm in half an hour. Time 15:51.

**Figure 5:**
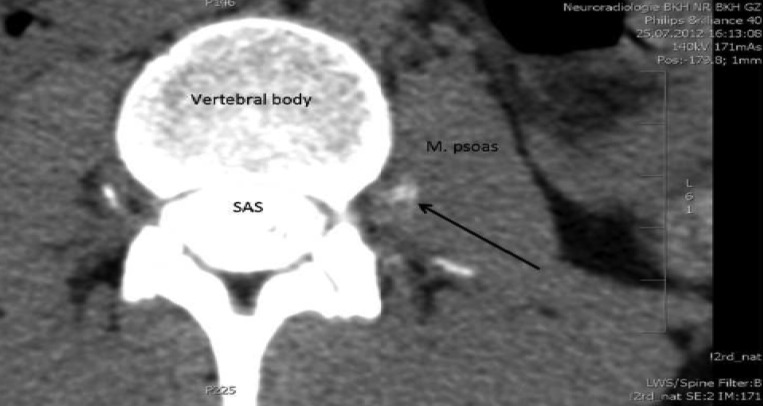
Transverse slice at the level of lumbar segment 3 showing the vertebral body and the nearby subarachnoid spaces (white) next to the psoas muscle (M. psoas) and the contrast agent (white) outside the subarachnoid spaces.

In a patient undergoing lumbar myelography we observed physiological CSF outflow at lower body nerves, near the injection site (Figure 1). The patient was examined for clinical reasons unrelated to this study. There was immediate distribution of the contrast medium throughout the neighboring SASs, at all the neighboring nerve roots (Figure 2), and down the respective peripheral nerves (Figure 3-5). The cause of this CSF outflow cannot be increased intrathecal pressure from injection since we observed a similar finding in many cases before. Also, this can be excluded on the basis of logical considerations since after injection of the contrast agent into the SAS the intrathecal pressure may at best increase for a few seconds, because injection was performed slowly and an equivalent volume of CSF was removed before injection. Apparently, the contrast agent (now dissolved in the CSF) follows the natural course of CSF outflow along the nerves. The pattern of distribution is not compatible with a diffuse distribution or absorption from the SAS into the neighboring tissues, but with the CSF outflow scenario as proposed by the PCOP hypothesis (6). Although our neuroradiological pictures do not allow us to exactly define the anatomical borders of the outflow pathway and determine whether these borders definitely represent the epineurium and perineurium, they support such assumption. We observed that CSF roughly began to distribute from the SASs into the neighboring psoas muscle following the course of the nerve through the tissues. The outflow velocity was about 50 mm per 30 minutes (Figure 4). In addition, CSF slightly and diffusely distributed into the tissues closely adjacent to the nerve at many sites along its outflow course (Figure 4,5). Such disappearance of contrast agent might be explained by diffusion through the natural borders, ie, nerve sheets, or alternatively may be produced by multiple small wind ups of the PCOP all along the nerve, ie, at multiple nerve muscle end plates. One should note that the anatomy of the wind up of the PCOP pathway has not yet been described in histological detail.

## OUTLOOK

There are still no studies dealing with CSF signaling at the PCOP sites, possibly mediated by various pathogenic CSF contents including solutes, microparticles, microvesicles, and cells. Our case is a rather typical finding, representing on overall CSF outflow velocity in still position of about 10 cm per hour. More conclusive and systematic studies are needed to more precisely determine the CSF outflow volumes and flow velocity along the PCOP, and interpersonal, intrapersonal, and local variability of CSF outflow. It is also important to define possible modulating factors of CSF outflow like age, sex, body mass, or the influence of motor activity. These aspects require systematic studies in animals and in humans.

Given the poorly explained mechanisms of pain generation in multiple sclerosis or experimental allergic encephalitis (19), or in experiments on pain hypersensibility by Levy et al (12), the PCOP-associated pain pathomechanisms mediated through pathogenic CSF contents should be further investigated. The widespread mast cell degranulation especially at the meninges observed in the experiments by Levy et al could be similar to LLNI or classical neuroinflammatory mechanisms (4).

Another question of interest was the overall balance between CSF production and CSF absorption outflow, the latter involving the outflow along the PCOP, glymphatic pathway (20), absorption at arachnoid granulations, and the balance of fluid exchange between the brain and SAS (21).

Also, it would be interesting to examine the complex CSF flow patterns involving the pulsatile brain (22,23) as the pulsatile brain drives volume transmission, which is balancing wiring transmission and is important for CSF signaling (7). The so called “roamer type” CSF signaling, which involves exosomes and microparticles (13), may help to better understand the unsolved questions and pathomechanisms in psychiatric and neurological disorders.
